# The Failure of Screening and Treating as a Malaria Elimination Strategy

**DOI:** 10.1371/journal.pmed.1001595

**Published:** 2014-01-28

**Authors:** Lorenz von Seidlein

**Affiliations:** Global and Tropical Health Division, Menzies School of Health Research, Casuarina, Northern Territory, Australia

## Abstract

Lorenz von Seidlein discusses the study by Katherine Halliday and colleagues and explores the reasons why a school-based screening and treatment strategy for malaria might have failed.

*Please see later in the article for the Editors' Summary*

**Linked Research Article**

This Perspective discusses the following new study published in *PLOS Medicine*:

Halliday KE, Okello G, Turner EL, Njagi K, Mcharo C, et al. (2014) Impact of Intermittent Screening and Treatment for Malaria among School Children in Kenya: A Cluster Randomised Trial. PLoS Med 11(1): e1001594. doi:10.1371/journal.pmed.1001594

Katherine Halliday and colleagues conducted a cluster randomized controlled trial in Kenyan school children in an area of low to moderate malaria transmission to investigate the effect of intermittent screening and treatment of malaria on health and education.

## Introduction

Falciparum malaria incidence is declining in many countries including in the formerly hyperendemic regions of sub-Saharan Africa [1]. Malaria control and elimination efforts continue to optimise the case management of patients as they present for health care whilst investigators search for a more aggressive approach that is feasible and effective. One strategy is to routinely screen the population and treat those who are infected, irrespective of whether they have symptoms or not. This strategy has been recommended by international health organizations on the basis of expert opinion and models [2].

## The Trial

In this issue of *PLOS Medicine*, Halliday and colleagues describe a trial to evaluate intermittent screening and treatment in schools [3] With malaria elimination in mind and guided by mathematical models, the investigators screened school children with rapid diagnostic tests (RDTs) and treated them with an appropriate antimalarial drug combination (artemether-lumefantrine) if they were RDT-positive. To evaluate the potential benefit of such an intervention, schools in the area were randomised to either receive or not receive the screening and treatment programme. The trial was conducted in 101 schools in the most southern part of Kenya where the country borders on Tanzania and the Indian Ocean. Over a 24-month period the investigators followed the study participants measuring health (e.g., anaemia and parasitaemia) and educational (e.g., attention span) indices. The findings were overwhelmingly disappointing. There were no health benefits among children in the schools with regularly screened and treated participants. If there was any effect on learning it was a negative one; a predefined sub-group of children in the intervention arm performed worse in their educational assessment than children in the control group.

## Why Did the Intervention Fail?

The results cannot be attributed to methodological uncertainty, a frequent cause of negative study outcomes, since the trial was conducted in a large sample of schools according to the highest procedural standards. There was excellent follow-up of and adherence by study participants. Most likely, children found to be parasitaemic did benefit from early treatment but this outcome was not measured, as the investigators were interested in school-based rather than individual-based effects. Why did these individual effects not translate to school-wide outcomes? First investigators didn't treat all infections, rather only those of a sufficiently high density detectable by RDTs. Any child with a parasitaemia with a density below the RDT's detection threshold on that day remained untreated. The dimensions of this reservoir of infections with densities below the RDT's detection threshold are incompletely understood and are currently under investigation using new molecular tools. RDTs may require a parasite density of 100,000 parasites/ml [4] to become positive. In contrast, PCR on 1 ml of blood can detect as few as 20 parasites/ml. The turn-around time for such complex molecular tests is currently measured in weeks rather than days and infections with parasite densities below 20 parasites/ml may still remain undetected. Second, and perhaps even more importantly, the majority of infections are contracted outside of school hours with children being bitten by mosquitoes late in the day and early in the morning in their homes. The prevention of re-infections would be crucial for a reduction in malaria burden.

Would the strategy have worked had the investigator screened and treated whole villages instead of school classes? Investigators in Burkina Faso have recently addressed precisely this question in a cluster randomised trial [5]. The investigators screened nine villages with RDTs, treated the participants who tested positive with an appropriate antimalarial drug combination (artemether-lumefantrine), and compared the findings with nine control villages. Just like in the study in Kenya, the health benefits were evaluated in terms of reduction in anaemia and parasitaemia, and just like in the Kenyan setting, no benefit of the intervention could be detected. The findings from these two studies suggest that as long as a subclinical malaria reservoir persists, malaria transmission will continue and a substantial reduction in parasite prevalence, not to mention malaria elimination, is unlikely. Since the currently available screening methods do not allow for timely detection and treatment, malaria elimination strategies based on screening and treating are doomed. Negative studies often go unpublished but these studies can often provide critical empirical evidence; in this case, evidence that malaria control resources should be invested into the search for alternative strategies to screening and treating.

## Using Models to Predict Outcomes

The evaluation of malaria elimination strategies requires considerable resources. Trials evaluating interventions to interrupt malaria transmission require populations living in a discrete geographic entity such as a village as unit of inference. To randomise villages in a statistically meaningful fashion involves tens of thousands of participants. Considering the time and resources required for such large undertakings, researchers use mathematical modelling to predict the outcome of interventions. Such models have been refined for more than a century and their popularity keeps growing despite the inherent complexity of vector borne diseases [6]. The investigators of the studies in Kenya, as well as in Burkina Faso, consulted mathematical models based on work by Okell and co-workers [7], which has been further adapted to predict the benefit of interventions including screening and treating [8],[9]. There is a range of potential reasons why the forecasts didn't hold up to the reality tests, likely related to the underlying assumptions of the models. Weather forecasts have become steadily more reliable as models are recalibrated and revised and this should also hold true for malaria transmission models. However for weather there is a continuous flow of data, while the same can't be said for data on malaria transmission. To avoid future disappointment, investigators are well advised to be aware of some of the more unrealistic assumptions and the limitations of the model they are consulting.

## The Future: Alternative Approaches

It is easy to criticise retrospectively the failure of well-intended interventions and infinitely harder to predict success. But some of the more successful interventions could guide investigators and policymakers towards more promising strategies especially during an emergency. Increasing the coverage of populations at risk with effective interventions (e.g., appropriate case management and long lasting impregnated bed nets) should lead to the elimination and ultimately the eradication of falciparum malaria in the decades to come. That is if the pathogens and the vectors weren't constantly evolving. Mosquitoes resistant to the available insecticides are spreading through sub-Saharan Africa and Asia [10]. *Plasmodium falciparum* strains resistant to artemisinins are spreading through Southeast Asia [11]. These two developments make a reversal of advances in malaria control a distinct possibility. The only way to stop the transmission of artemisinin resistant strains immediately and hence delay the spread to the African continent is to interrupt the transmission in targeted areas. To achieve targeted malaria elimination rapidly the reservoir of sub-microscopic infections has to be eliminated, which requires the presumptive treatment irrespective of signs, symptoms, or test results of the targeted populations. Resistance to this approach is considerable. Exposing potentially uninfected people to repeated treatment courses is unacceptable to many public health experts as well as some members of the target population. Yet there are examples of places where this strategy has worked. For example, Kaneko and colleagues eliminated malaria with a programme that included multiple rounds of mass drug administrations from Pacific islands [12], but the generalizability of a successful intervention on islands has been questioned. The world's largest population, the People's Republic of China, is close to eliminating all malarias [13]. Their strategy relies heavily on the presumptive treatment of large populations, which becomes more targeted over time as malaria incidence decreases. Their approach has not been evaluated in randomised trials along the paradigm of evidence-based science. But after other approaches have failed perhaps an evaluation of strategies based on presumptive treatment of targeted populations should now have the highest priority?
